# Large Cell Neuroendocrine Cervical Carcinoma: A Case Report

**DOI:** 10.7759/cureus.6544

**Published:** 2020-01-02

**Authors:** Humaira Sarfraz, Samridhi Syal, Sara Sarfraz, Stephanie Fuentes Rojas, Ejaz Janjua

**Affiliations:** 1 Internal Medicine, Houston Methodist Hospital, Houston, USA; 2 Medicine, Combined Military Hospital Lahore Medical College, Lahore, PAK

**Keywords:** large cell neuroendocrine carcinoma, cervical cancer

## Abstract

We herein report a unique case of a large cell neuroendocrine tumor in a female presenting with right upper quadrant pain. She was found to have multiple metastatic lesions in the liver noted on imaging and underwent workup for malignancy of unknown origin. The initial differential diagnoses included gastrointestinal, urothelial, genital, or breast primary sites. The cervical biopsy results were consistent with large cell neuroendocrine tumor, and the patient was subsequently started on chemotherapy.

## Introduction

Neuroendocrine tumors of the uterine cervix are a group of rare malignancies characterized as aggressive and prone to early metastasis. They account for only 5% of all cervical cancers [1]. Large cell neuroendocrine carcinomas (LCNC) are an even more uncommon occurrence with almost 70 cases reported to date [2]. Furthermore, the histological criteria for cervical LCNC include the presence of large cells with abundant cytoplasm, vesicular nuclei with prominent nucleoli, and a high mitotic rate. The growth pattern of LCNC is an insular, organoid, trabecular, or solid pattern, often with peripheral palisading or rosette formation, and focal tumor necrosis. The immunohistochemical neuroendocrine markers include synaptophysin, chromogranin, CD56, and neuron-specific enolase (NSE). The immunohistochemistry-positive rates in LCNC are 87% for chromogranin, 56% for synaptophysin, and 88% for at least one of chromogranin, synaptophysin, or NSE [3]. This report describes a rare presentation of large cell neuroendocrine carcinoma.

## Case presentation

**Figure 1:**
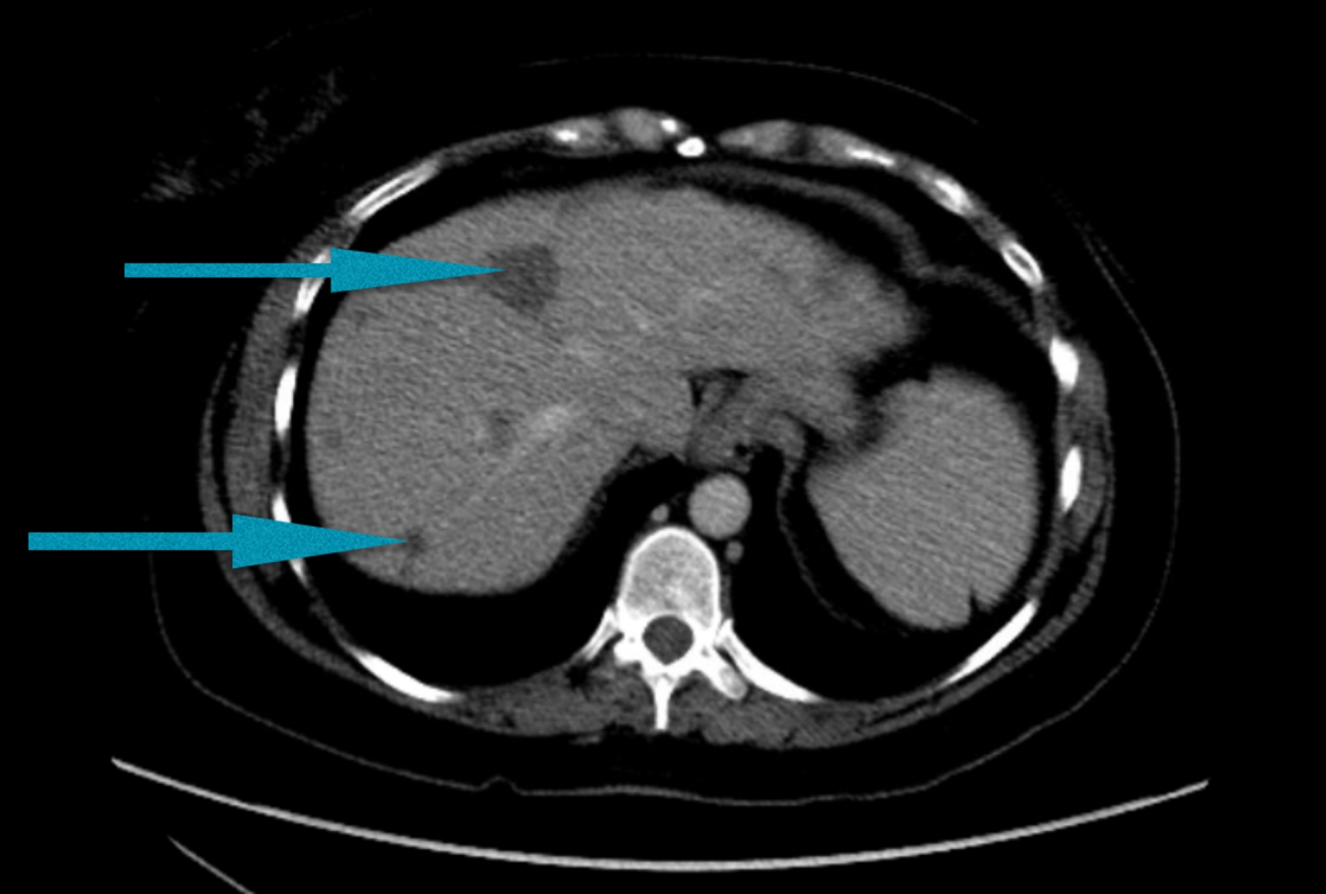
Multiple metastases to the liver shown by blue arrowheads on a computed tomography scan of the abdomen and pelvis

**Figure 2:**
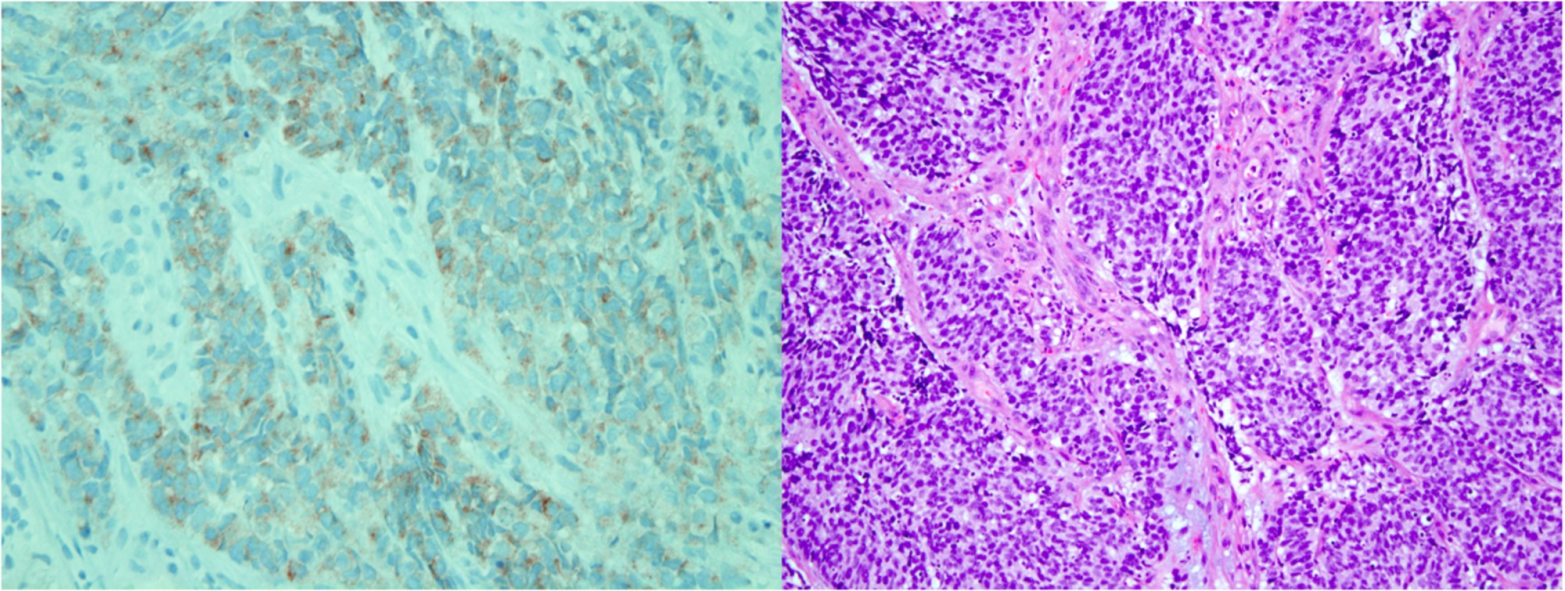
Large cell neuroendocrine carcinoma. The image on the left depicts sheets of oval and round cells with abundant cytoplasm. There was brisk mitotic activity and occasional nucleoli present. On the right, synaptophysin staining of the cervical mass was congruent with neuroendocrine carcinoma.

A 40-year-old woman with a history of hypertension presented to an outside hospital with right upper quadrant abdominal pain. Computed tomography (CT) of the abdomen/pelvis revealed multiple masses ranging in size from 1 to 7 cm, almost replacing the liver (Figure 1). It also showed a right external iliac chain lymphadenopathy. She, therefore, underwent an ultrasound-guided liver biopsy that showed poorly differentiated carcinoma, likely small cell carcinoma. She was subsequently transferred to our facility with a diagnosis of metastasis of unknown primary origin for further evaluation. On presentation to our facility, the patient continued to have right upper abdominal pain. She denied fever, night sweats, cough, hemoptysis, shortness of breath, flushing, diarrhea, or lymphadenopathy. She reported heartburn, and while she denied melena or hematochezia, she reported intermittent hematuria over the past few months before admission. Additionally, while menses were typically regular, during admission, she noted spotting which she never had in the past. Family history was non-contributory. Social history was significant for former smoking: about 10 cigarettes per week for three years 10 years before admission. Regarding her cancer screening, she had no colonoscopy, no mammogram, and last pap smear was 16 years prior to admission. On physical exam, her temperature was 98.3°​​​​​​​F, blood pressure 117/85, heart rate 78 breaths per minute (bpm), respiration rate​​​​​​​ 20/min, O_2_​​​​​​​ saturation 98% on room air, and a body mass index (BMI) of 27 kg/m^2^. The cardiovascular exam showed a normal rate and regular rhythm with no murmurs, rubs, or gallops. Her lungs were clear to auscultation bilaterally. The abdomen was soft, non-distended, normoactive bowel sounds, and severe tenderness to palpation on the right upper quadrant. On breast examination, there was no nipple discharge, masses, nodules, or axillary lymphadenopathy noted bilaterally. The pelvic exam revealed an 8 cm cervical mass that replaced the entire cervix with small necrotic-appearing areas and a small amount of dark red blood noted in the vaginal vault. Laboratory studies were significant for a cancer antigen (CA) 125 of 427, CA 19-9 of 89, alpha-fetoprotein​​​​​​​ (AFP) of 3.2, and carcinoembryonic antigen​​​​​​​ (CEA) of < 1.2. Other testing included a chest CT scan without any abnormalities, an unremarkable colonoscopy, and an esophagogastroduodenoscopy​​​​​​​ (EGD) which showed reflux esophagitis. She subsequently underwent a biopsy of the cervical mass for which pathology was consistent with large cell neuroendocrine carcinoma as seen in Figure 2. The biopsies showed an infiltrating carcinoma composed of sheets of oval and round cells with abundant cytoplasm. There were brisk mitotic activity and occasional nucleoli. Immunohistochemistry revealed patchy positivity for cytokeratin (CK) 5/6, strong and diffuse P16 positivity, and cluster of differentiation​​​​​​​ 56 (CD56) and chromogranin negativity but did show weak positivity of synaptophysin throughout the tumor.

Soon after the diagnosis of cancer was made, she was not deemed a surgical candidate, given the metastatic disease. Therefore, she was started on chemotherapy with cisplatin/etoposide. She received four cycles of chemotherapy with a partial response. Almost three months later, she was readmitted with progressive disease and worsening hepatic function. At that point, she was not even a candidate for chemotherapy, given the progressive hepatic dysfunction. The family elected for palliative care and the patient expired within days of that hospital admission.

## Discussion

This case presented as a challenge because the initial liver biopsy done at an outside facility showed immunohistochemistry staining positive only for CK7; a thyroid transcription factor 1 (TTF-1) was negative with negative neuroendocrine markers. Therefore, the initial differential diagnosis included gastrointestinal, urothelial, or breast as possible primary malignancies and importantly did not include neuroendocrine carcinomas. However, after careful review of the pathology slides with comparison to the ones from the cervical mass, it was determined that the primary site was indeed the cervix and the diagnosis of large cell neuroendocrine carcinoma was made. This case illustrates the importance of a thorough history/physical examination and regular cancer screening when evaluating a case of metastasis of an unknown primary origin. Most cases of a large neuroendocrine tumor of the cervix are diagnosed at an early stage with vaginal bleeding or during regular pelvic exams. Furthermore, given the report that LCNCs have been histologically been diagnosed as a mixed type, coexistent with a small cell neuroendocrine carcinoma, adenocarcinoma, or squamous cell carcinoma, this case highlights the importance of procuring an adequately-sized biopsy when possible to assure an accurate diagnosis [3]. Also, it would be interesting to check the human papillomavirus (HPV) status and impact on tumor behavior and response to treatment in patients with LCNC. This information was, unfortunately, not available for our patient. Grayson et al. found the HPV status to be positive in seven out of the 12 cases of large neuroendocrine tumors suggesting a potential association between the viral infection and this rather rare, yet aggressive tumor [4]. Hence, the vitality of routine clinical screening and clinicians being mindful of the differential is of supreme importance in the early detection and management of this malignancy. Another clinical feature to be noted in this case was the absence of the routine symptoms of flushing, shortness of breath, and diarrhea, which are commonly present in patients with metastatic neuroendocrine tumors. This suggests that while the presence of these features should raise our clinical suspicion higher, their absence does not rule out neuroendocrine tumors. 

Neuroendocrine cancers are aggressive tumors with high metastatic potential. They are frequently misdiagnosed and are generally associated with a poor prognosis [5]. Moreover, this is a rare diagnosis; hence, treatment options have majorly been adapted from management used for neuroendocrine tumors of the lung [6]. While the role of surgery in prolonging survival is controversial, chemotherapy is the main management option utilized against it. Most chemotherapy options available include carboplatin and etoposide (both of which were used in our patient). Other frequently used combinations include cyclophosphamide, doxorubicin, and vincristine. A multivariate analysis by Embry et al. showed that incorporation of chemotherapy at any point in cancer treatment was associated with prolonged survival [7]. However, an earlier disease stage was associated with a better survival; the mean overall survival period for stage IV cancer was noted to be 1.5 months. Our patient survived four months post-diagnosis.

## Conclusions

In conclusion, this case report sheds light on a rare, aggressive malignancy of the cervix, which is often misdiagnosed and associated with an unfavorable prognosis. Henceforth, we would like to highlight the importance of considering a neuroendocrine tumor as a differential when assessing for potential malignancies which could help to identify it and explore treatment options at an earlier stage.
